# Mouflon and Domestic Sheep Phylogeny: Ancestry, Domestication, and Evolutionary Dynamics

**DOI:** 10.3390/life15091446

**Published:** 2025-09-15

**Authors:** Paolo Mereu, Monica Pirastru, Fabio Scarpa, Marco Zedda, Luisa Bogliolo, Salvatore Naitana, Giovanni Giuseppe Leoni

**Affiliations:** 1 Dipartimento di Scienze Biomediche, Università degli Studi di Sassari, 07100 Sassari, Italy; fscarpa@uniss.it (F.S.); gioleoni@uniss.it (G.G.L.); 2 Dipartimento di Medicina Veterinaria, Università degli Studi di Sassari, 07100 Sassari, Italy; mzedda@uniss.it (M.Z.); lbogliolo@uniss.it (L.B.); snaitana@uniss.it (S.N.)

**Keywords:** mouflon genetics, sheep evolution, genetic introgression, hybridization, molecular taxonomy, genetic distinctiveness, *Ovis gmelini*, *Ovis aries*

## Abstract

The ancestry of domestic species from their closest wild relatives is one of the most debated and intriguing topics in evolutionary genetics. This review synthesizes current scientific understanding of the phylogenetic relationships between wild mouflon populations and domestic sheep (*Ovis aries*). It delves into the complex ancestry, tracing the primary role of the Asiatic mouflon (*Ovis gmelini*) as the progenitor, while also addressing the debated contributions of other wild *Ovis* species. The report explores the insights gained from diverse genetic markers, including mitochondrial DNA haplogroups and comprehensive whole-genome sequencing, highlighting their strengths, limitations, and the resolution of phylogenetic discrepancies. The multi-faceted taming process is examined, discussing proposed evolutionary mechanisms such as the domestication syndrome and thyroid hormone hypotheses, alongside human-mediated selection for key phenotypic traits like horn morphology, coat type, and tail characteristics. Furthermore, the pervasive role of hybridization and introgression between wild and domestic populations is analyzed, detailing its impact on genetic distinctiveness, adaptive potential, and the critical implications for conservation strategies. Finally, the review addresses ongoing scientific debates, particularly concerning the taxonomic classification of European mouflon, and identifies crucial avenues for future research to further unravel the intricate evolutionary tapestry of *Ovis* species. To ensure taxonomic consistency and promote conservation, nomenclature should be updated across all public repositories. Following the widely accepted classification that recognizes its lineage from the Asian mouflon, the Corsican and Sardinian mouflon should be designated as *Ovis gmelini musimon*.

## 1. Background: The History of Sheep and Mouflon

**Figure 1:**
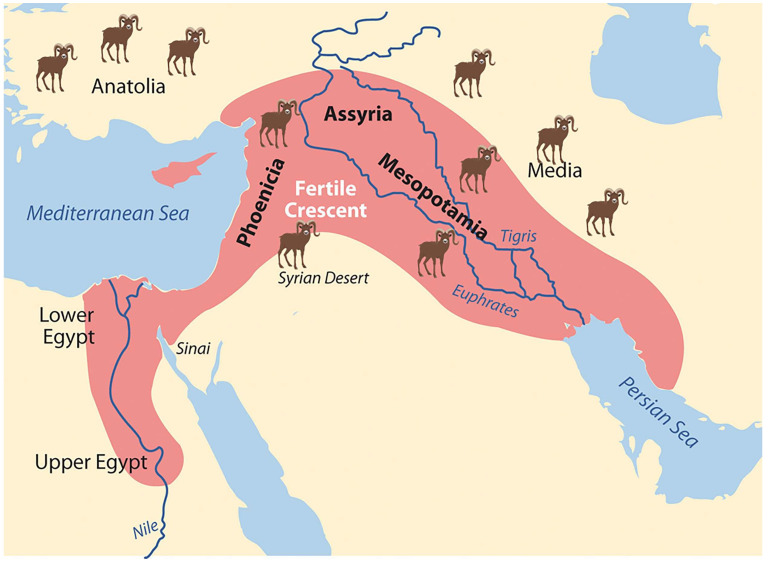
Geographical map indicating the most credited domestication centers for sheep.

Sheep are among the earliest animals domesticated by humans, with their history tracing back to 10,000 to 8000 years Before the Common Era (BCE) in a region encompassing Anatolia, ancient Mesopotamia, and Southwest Asia [1,2,3,4,5,6,7] (Figure 1).

This early domestication underscores the foundational importance of sheep in human history, as they have since represented a crucial resource globally, providing meat, wool, skin, milk, and other necessities to large populations worldwide [6,7,8]. Over time, domestic sheep were transported around the world, and today there are hundreds of domestic breeds alongside multiple wild sheep lineages [6,7,9,10].

Despite extensive research using molecular data from modern sheep lineages [8,11,12,13,14,15,16,17], key questions remain unresolved. These include the precise location and timing of sheep domestication, the identity of the wild progenitors involved, and the post-Neolithic demographic histories of domestic sheep populations. Earlier studies suggested multiple Asiatic sheep species as potential ancestors of domestic sheep, with urial (*Ovis vignei*; Blyth, 1841), argali (*Ovis ammon*; Linnaeus, 1758), and mouflon (*Ovis gmelini*; Blyth, 1841) considered as potential candidates [18,19]. However, subsequent evidence—mainly based on mitochondrial DNA (mtDNA) sequence analyses—has ruled out urial and argali, indicating the Asiatic mouflon (*O. gmelini*) as the primary maternal source of domestic sheep [8,16,20,21,22,23,24].

The Asiatic mouflon inhabits a vast area, including Armenia, southern Azerbaijan, Cyprus, and the northern, southern, central, and western regions of Iran, as well as the eastern and central regions of Turkey. Five subspecies have been identified based on morphological distinctions and geographic distributions [25,26]: the Armenian mouflon (*O. gmelini gmelini*; Blyth, 1841), Anatolian mouflon (*O. gmelini anatolica*; Valenciennes, 1856), Isfahan mouflon (*O. gmelini isphahanica*; Nasonov, 1910), Laristan mouflon (*O. gmelini laristanica*; Nasonov, 1909), and Cyprian mouflon (*O. gmelini ophion*; Blyth, 1841). The intricate intraspecies taxonomy of the Asiatic mouflon has led to uncertainty regarding the genetic contributions of these various subspecies to the domestication of sheep [6,27,28].

The hypothesis suggesting the Asiatic mouflon as the domestic sheep’s primary ancestor has not been fully confirmed. The genetic relationships between various mouflon subspecies (e.g., Anatolian, Cyprian, Iranian) and domestic sheep remain unclear. This uncertainty reflects the broader scientific debate surrounding the topic of the exact lineages and the extent of genetic contributions from wild ancestors to modern domestic sheep. Such a complexity arises from several factors, including the presence of multiple potential ancestral sources, historical introgression events, and the challenges of reconciling results from different genetic markers [7,8,29,30,31]. Genetic studies based on retrovirus integration and genome-wide data suggest that early domestic flocks were genetically diverse [2,6]. These flocks likely underwent multiple migrations and hybridization events before giving rise to modern sheep populations.

The genetic structures of modern domestic sheep cluster into two main geographic groups: European and Asian/African. This east–west genetic split has been traced back to 7000–6000 BCE, suggesting their early diversification. In addition, based on the analysis of ancient samples, modern breeds show higher genetic affinity to each other than to Neolithic sheep, implying significant post-Neolithic admixture among sheep populations [6]. This admixture may have involved both introgression from wild sheep and the spread of sheep with desirable traits across continents [12,32,33,34,35]. Indeed, introgressions from wild relatives into domestic sheep populations [12,13,32,36] have allowed sheep to adapt to different environments and spread throughout the world, undergoing genetic improvements in various production systems. As a result, numerous unique breeds have developed [11,32], and the extensive variations observed in both landraces and improved breeds underscore the genomic diversity, adaptive characteristics, and important agronomic traits present in domestic sheep [15,37,38].

Based on the results reported above, the domestic sheep’s origin involved a two-step process: initial domestication and early divergence, followed by extensive post-Neolithic diversification. The early timing of sheep domestication suggests an initial phase that may have focused on protection and ensuring a constant food source, with minimal human interaction or artificial selection, rather than immediate intensive breeding for specific traits [1,2,23,39]. This contrasts with later, more deliberate selection observed in modern breeds [38,40,41,42]. It is crucial to understand this primitive phase of sheep domestication in order to distinguish between genetic changes that existed prior to domestication and those that are directly attributable to human-mediated selection. This historical context reveals domestication not as a singular event but as a continuous process characterized by the gradual accumulation of genetic, phenotypic, and physiological changes [1,7,43].

This review aims to elucidate the intricate phylogenetic relationships between mouflon and domestic sheep, emphasizing the Asiatic mouflon as the primary wild ancestor and highlighting the profound impact of domestication and subsequent genetic exchange involving wild, feral, and domestic sheep. While significant progress has been made through mitochondrial and whole-genome sequencing studies, particularly in identifying distinct haplogroups and introgression events, ongoing debates persist regarding taxonomic classification and the precise nature of domestication. The continuous genetic admixture between wild mouflon and domestic/feral sheep poses a critical conservation challenge, necessitating vigilant monitoring and management strategies.

## 2. Taxonomic Status and Ancestral Lineages of Mouflon

Mouflon (*O. gmelini*) is a wild sheep species known for its strikingly curved horns and a characteristic reddish-brown coat [7,30,44]. Its natural range spans Central Asia, from Turkey in the west to Pakistan in the east, typically occupying mountainous regions up to 3000 m above sea level [26]. However, mouflon populations were also introduced early in European forested areas [13,45,46,47]. Notably, the Mediterranean islands of Sardinia and Corsica host ancient mouflon populations descended from wild Neolithic ancestors that first arrived on these islands approximately 7000 years ago [5,39,48].

The status of the European mouflon is particularly contentious. Although it is generally considered a subspecies within the Asiatic mouflon clade [9,23], its origin and “wild” status have been subjects of considerable debate [2,20]. Historically, European mouflons, especially those found on Corsica and Sardinia, were thought to be remnants of an ancient European wild sheep population [49]. However, archaeological evidence suggests that these populations were introduced by humans to Cyprus in the Early Neolithic period (approximately 10,000 years ago) and subsequently to Corsica and Sardinia [10,50]. It is assumed that these populations were far from being domesticated in the modern sense but were merely kept in fenced areas to protect them from predators and to provide a constant, easily accessible source of meat and skins, with minimal interaction between humans and animals and no artificial selection [1,7,9,39]. Consequently, their classification as truly wild or feral animals remains a point of scientific discussion.

**Figure 2:**
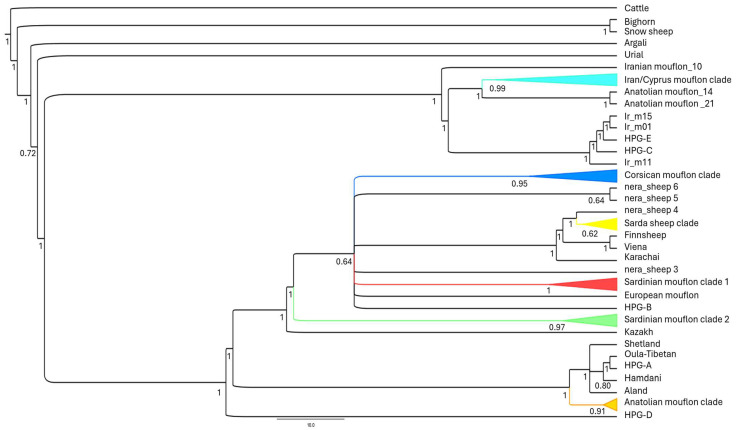
Bayesian tree analysis calculated using MrBayes v. 3.2.4 [51] and based on the whole mitogenome sequences highlighting the main splitting events within the mouflon/sheep group and the phylogenetic distinctiveness of the Corsican and Sardinian mouflon. Two independent runs, each consisting of four Metropolis-coupled reversible-jump Markov chain Monte Carlo chains, were performed. Analyses were carried out for 10 million generations and the trees were sampled every 10 generations. Convergence of chains was checked by ensuring that the standard deviation of split frequencies reached and stabilized at a value <0.01. For each run, the first 25% of sampled trees were discarded.

This taxonomic ambiguity extends to the systematic classification and nomenclature of Corsican and Sardinian mouflons. In many scientific databases and websites, such as the National Center for Biotechnology Information (NCBI) (https://www.ncbi.nlm.nih.gov/genbank/, accessed on 9 July 2025) and the CABI Digital Library (https://www.cabidigitallibrary.org/doi/full/10.1079/cabicompendium.71353, accessed on 9 July 2025), they are currently classified as a subspecies of domestic sheep (*O. aries musimon*). Phylogenetic studies based on the analysis of whole mitogenomes advocate for a revision of this classification [5,23], proposing their re-designation as a subspecies of their wild relatives (*O. gmelini musimon*). This proposed change is not merely an academic exercise; it is supported by evidence of their phylogenetic distinctiveness and basal lineages, which are associated with the early European expansion of the first sheep (see Figure 2 from [23] for further details) (Figure 2).

Such a reclassification would align their nomenclature with that of the Cyprian mouflon (*O. gmelini ophion*) and Anatolian mouflon (*O. gmelini anatolica*), which are already recognized as subspecies of the wild *Ovis gmelini* [26]. This debate reflects a complex history where early domesticates might have returned to a feral state or interbred with existing wild populations, blurring the line between truly wild and domestic lineages [33].

Reclassifying these populations to recognize their distinct wild genetic heritage is a critical step to facilitate their conservation, especially given the ongoing threat of hybridization with feral domestic sheep. This situation underscores the dynamic nature of domestication, a process that is not a one-way street, and highlights the need for a flexible and genetically informed approach to taxonomy and conservation.

## 3. Genetic Contribution from Other Wild Sheep

While the Asiatic mouflon is widely accepted as the most credited ancestor of domestic sheep, the potential role of other wild *Ovis* species has also been investigated. The urial was historically considered a possible progenitor of domestic sheep, particularly given its occasional interbreeding with mouflon in the Alborz Mountains of northern Iran and the Kavir Desert of north-central Iran [52]. Several molecular studies, notably those based on mtDNA, have found no evidence for significant maternal contributions from urial and argali to the main domestic sheep lineages [3,7,18,27]. However, more recent genomic analyses, with their improved resolution, indicate rare but significant introgression events from both urial and argali into domestic sheep. For instance, the *VPS13B* gene shows introgression signals from urial and/or mouflon [24]. Similarly, the argali has been suggested to have introduced alleles into Southeast Asian domestic sheep lineages, with the introgression of the *MSRB3* gene which has been found to be specifically associated with variations in ear morphology [24]. The shift in understanding from viewing these species as direct ancestors to recognizing them as sources of introgression reflects the increasing resolution of genetic studies. Early mtDNA studies primarily trace maternal lineages and major foundational events, whereas whole-genome sequencing can detect more subtle, localized gene flow that occurred after the initial domestication, contributing specific adaptive or morphological traits.

## 4. Genetic Architectures Unravelling Phylogeny

### 4.1. Insights from mtDNA Studies

Mitochondrial DNA is a powerful tool for phylogenetic analyses due to its unique characteristics: it is inherited exclusively through maternal lineages and evolves approximately five to ten times faster than nuclear DNA [53]. Its clonal inheritance and near-neutral selection make it particularly well-suited for investigating phylogenetic relationships, tracing maternal origins, and estimating evolutionary divergence times [54,55].

Early studies of the sheep mtDNA control region polymorphism identified seven haplogroups (HPGs), though only five of them (A, B, C, D, E) have been detected in modern domestic breeds and confirmed by whole mitogenome sequences analyses, with HPGs F and G having disappeared [29] (Table 1).

- HPGs A and B: These were the first haplogroups identified, broadly corresponding to Asian and European origins, respectively [18]. HPG-A is commonly found in Central Asian and some European domestic sheep. Within HPG-A, Anatolian mouflons are basal to domestic sheep, suggesting a potential role in sheep domestication [7,30]. HPG-B, widespread across Eurasia, is predominant in European domestic sheep and exhibits close resemblance to European mouflon haplotypes [3,7,18,48]. Corsican and Sardinian mouflons clustered within HPG-B, which is associated with the early Neolithic colonization of Europe, and their phylogenetic distinctiveness suggests they are remnants of early pre-domestic ancestors that returned to the wild after the introduction of more economically valuable woolly sheep [3,5,39]. Indeed, the Corsican and Sardinian mouflon lineages within HPG-B originated between 120,000 and 80,000 years ago, evolving separately from domestic sheep breeds [23].
- HPG C: Recognized as the third major phylogenetic branch, HPG-C has been detected at low frequencies in Portuguese native sheep and in individuals from the Caucasus, Middle East, and Asia [56,57]. Maternal HPG-C, along with HPG-E, is believed to have originated from Asiatic mouflon populations in Iran [17,23].
- HPG D: This fourth maternal lineage, mainly found in domestic sheep from Caucasus region and Turkey [58,59], is considered the closest to the Anatolian mouflon (*O. gmelini anatolica*) [16,56].
- HPG E: The fifth haplogroup identified through D-loop, CytB, and whole-mitogenome sequences analyses is a key mitochondrial haplogroup of Iranian Asiatic mouflon [23,31].
- HPG X: This wild haplogroup is found in Cyprian, Anatolian, and Iranian mouflons and appears to be basal to the domestic C and E haplogroups [23,60].

The presence of these diverse mitochondrial lineages at the onset of sheep domestication suggests that the rise of current mitochondrial lineages predates the domestication process [5,23]. This implies that domestication did not create new major maternal lineages but rather recruited existing, highly divergent wild lineages. Such findings support the idea that early domestication was probably a less controlled process involving the incorporation of available wild stock, rather than intensive breeding from a single, uniform ancestral group. Consequently, the genetic diversity observed in modern sheep partly reflects ancient wild diversity, not solely a product of post-domestication mutation or selection. This challenges simplistic models of domestication as a singular event from a homogeneous ancestral population, instead pointing to a more complex scenario involving the recruitment of diverse wild genetic backgrounds. The early radiation that originated the current variability within the domestic sheep and mouflon clade is estimated to have occurred approximately 810,000 years ago [23].

### 4.2. Contributions from Nuclear DNA and Whole-Genome Sequencing

Whole-genome sequencing (WGS) represented a significant advancement in genetic research, providing comprehensive data that can resolve complex population structures, assess genetic diversity, and reconstruct phylogenetic relationships with a level of detail unattainable with uniparental markers. This technology allows for the detection of subtle introgression events and the precise association of specific genes with phenotypic traits [61,62]. Recently, Anatolian and Cyprian mouflons were suggested as the closest wild relatives of domestic sheep [30]. These two Asiatic mouflon subspecies indeed show higher genetic affinity with domestic sheep than all other studied wild sheep genomes, including the Iranian mouflon (*O. gmelini*). Another study provided evidence that sheep domestication likely occurred in Central Anatolia, outside the traditionally assumed Fertile Crescent [6]. The genetic similarity of Anatolian Epipaleolithic sheep and modern Anatolian and Cyprian mouflons to domestic sheep supports such a hypothesis. However, it remains unclear whether sheep domestication involved a unique center or multiple centers, as reported for other livestock species [63,64,65]. In addition, the study from Kaptan and coll. Ref. [6], detected early admixture in Central Anatolia during the Neolithic and a shared ancestry in modern sheep breeds that was not observed in ancient genomes, suggesting recent introgression events within the last two millennia, potentially driven by selection of positive and desired traits [6,66].

More recent WGS analysis of Asiatic mouflon populations in Iran has identified two distinct subpopulations: *O. gmelini_2*, geographically restricted to Kaboodan Island within Urmia Lake National Park, and *O. gmelini_1*, distributed over a broader area [31]. This ability of high-resolution genomic data to pinpoint specific ancestral populations as sources of genetic material for particular domestic sheep breeds goes beyond the generic origin of “Asiatic mouflon”. It identifies specific geographic and subpopulation contributions, which is crucial for understanding the precise routes and timings of domestication and subsequent gene flow. This level of detail is essential for tracing the fine-scale geographical origins of domestic animals and understanding how specific wild populations contributed to the genetic diversity and adaptive traits of modern breeds, moving from a broad brushstroke of ancestry to a detailed map of genetic exchange.

Analysis of 72 whole-genome sequences from domestic sheep and mouflon suggested that European mouflon likely originated through hybridization between a now-extinct sheep species in Europe and feral domesticated sheep around 6000–5000 years ago. In contrast, the Asiatic mouflon diverged from domestic sheep approximately 12,800–8800 years ago, coinciding with the domestication process [8]. According to the results of this study, sheep were domesticated from Asiatic mouflon in the Fertile Crescent ~12,000–10,000 years ago during the Neolithic agricultural revolution, and the domestication led to a bottleneck in genetic diversity, with domestic sheep showing lower genomic diversity when compared to Asiatic mouflon.

### 4.3. Discordance in Reconstructing Phylogeny Between mtDNA and nuDNA

A significant ongoing debate in the field concerns the discrepancies observed between phylogenetic reconstructions inferred from different genetic markers [67,68]. For example, mitogenome-based analysis suggests that the Cyprian mouflon is more closely related to the Iranian mouflon [23], while whole-genome sequencing analysis places the Cyprian mouflon closer to the Anatolian mouflon [30]. This divergence stems from fundamental differences between mtDNA and nuclear DNA (nuDNA). MtDNA, being clonally inherited, traces only the maternal lineage, providing a clear, non-recombining phylogenetic signal useful for deep divergence. NuDNA, on the other hand, is biparentally inherited and subject to recombination, offering a more comprehensive picture of the overall genomic history. MtDNA analyses can be influenced by factors such as effective population size, incomplete lineage sorting (where ancestral polymorphisms persist and sort differently in descendant lineages), and sex-biased dispersal (e.g., male-mediated gene flow) [67]. The fragmentation of early populations, for instance, could lead to different mtDNA haplotypes becoming fixed in geographically isolated groups, even while nuclear gene flow continues. Such discrepancies are not necessarily errors but provide valuable insights into specific evolutionary processes, such as sex-biased migration or historical demographic events, that differentially affect maternal versus overall genomic inheritance. This highlights that a complete understanding of phylogeny necessitates integrating data from multiple genomic sources.

## 5. Domestication and Selection Signatures in Domestic Sheep

Domestication is characterized by the gradual accumulation of genetic, phenotypic, and physiological differences between wild and domesticated species [7,69]. The process is understood as a multi-stage evolutionary trajectory. According to Wilkins et al. [70], the initial phase, driven by selection for tameness, might have indirectly led to the establishment of a suite of phenotypic traits, a process known as “domestication syndrome”. This concept, first highlighted by Darwin and formalized by Wilkins et al. [70], postulates that selection for docility indirectly influences traits that are seemingly unrelated to tameness. These domestication-syndrome-associated traits are often linked to changes in the formation, differentiation, or migration patterns of NCCs, which are crucial for regulating cranial NCC fate, migration, and proliferation [71,72]. An alternative, yet not mutually exclusive, explanation is the “thyroid hormone hypothesis”, proposed by Crockford [73,74], which relates the domesticated phenotype to altered thyroid-hormone-based signaling [75]. While these initial, often unconscious, selections laid the foundation, subsequent and more deliberate human-mediated targeted selection played a critical role in refining specific domestic phenotypes for increased productivity and utility. This layered evolutionary history suggests that broad, early changes were later fine-tuned to meet specific human needs. Such a perspective moves beyond a simplistic view of “humans domesticated sheep” towards a nuanced understanding of how biological predispositions (e.g., social behavior, manageable size, early sexual maturity, high reproductive rates) interacted with different phases and intensities of human selection over millennia, resulting in the complex genetic and phenotypic landscape of domestic sheep [76].

The transition from wild mouflon ancestors to modern domestic sheep involved major phenotypic changes. Wild ancestors typically possessed horns in both sexes, coarse hair, and specific color patterns, which gradually evolved into the mostly polled (hornless), woolly, and white sheep breeds common today [7,44]. These transformations reveal important aspects of the selective pressures at play during domestication.

- Horn morphology: Wild sheep typically possess horns, which are crucial for intra-sexual competition [7,44,77]. In contrast, domestic sheep frequently exhibit polledness (the absence of horns), a trait preferred by farmers for safety and ease of management, as it avoids the need for dehorning [78]. The *RXFP2* gene has been identified as playing a prominent role in the presence or absence of horns, and introgression of an *RXFP2* haplotype from Iranian mouflon is associated with the spiral horn trait in domestic sheep [24]. Based on a recent molecular study, polledness is associated with a 1.8 kb insertion in the 3′ UTR region of the *RXFP2* gene, but this variant does not fully explain the variability in horn status across breeds [78]. The *Wnt* signaling pathway is also implicated in horn formation, linking this trait to the neural crest cell hypothesis [79].
- Coat type and color: A key transformation was the shift from annual wool shedding in wild mouflon to continuous fleece growth in domestic sheep, a trait likely selected for its utility in wool production for clothing [7,43]. NCCs are hypothesized to be involved in follicle morphogenesis [80] and the structural changes of the coat [81]. The variation in coat color, often white in domestic sheep due to artificial selection, is a complex trait influenced by genes such as extension (*E* gene), *ASIP* (or agouti), and *POMC*, which regulate melanin production [82,83,84,85].
- Tail length and fat deposition: The short, thin tail of mouflon suggests that the diverse tail phenotypes observed in domestic sheep emerged later in the domestication process [41]. The development of a fat tail, for instance, is considered an adaptive response to climate change, with ancient breeders selecting fat-tailed sheep for their enhanced adaptability to desert conditions and as a valuable source of fat [34,86]. Genes like bone morphogenetic protein 2 (*BMP-2*) and platelet-derived growth factor D (*PDGF-D*) are potential causative genes for tail phenotype and fat deposition [35,87]. The T-box transcription factor T gene (*TBXT*) is also involved in regulating tail length in mammals, including sheep [41]. The CT/CT genotype of *TBXT*:c. [333G>C; 334G>T] was found exclusively in fat-rumped sheep and is significantly associated with the tailless phenotype. This genotype was absent from long-tailed and short-tailed breeds [88]. The emergence of these phenotypic changes is not random but represents direct responses to human utility and environmental adaptation, highlighting the strong selective pressures exerted during domestication. Analyzing these phenotypic shifts, coupled with the identification of underlying genes, offers powerful insights into the genetic architecture of domestication and how human needs and environmental factors shaped the evolution of an entire species. It also suggests that some traits, like long tails, might have been coselected without immediate selective advantages [7,41].

A recent study investigated the selection signatures among five meat sheep breeds, including Charmoise, Charollais, Lacaune, Suffolk, and Texel [38]. Although all breeds have been subjected to selection programs aimed at improving meat production and quality traits, the direction and intensity of selection have varied significantly and each breed exhibited unique genomic regions under selection, which involved:

- in the Charmoise sheep breed, genes linked to carcass traits like bone percentage, lean meat yield, and hot carcass weight (*HOXD3*, *MAP3K20*, *RAPGEF4*, *SP3*, *LPGAT1*, *DNAH8*, *KIF6*, and *ABCD3*);
- in Charollais, genes associated with body weight, bone density, and muscle weight in carcass (*DGKB*, *ADGRB3*, and *PITPNC1*);
- in Lacaune, genes related to growth traits like average daily gain, body weight, and bone density (*ANO4*, *GRID2*, and *DBF4B*);
- in Suffolk, genes linked to carcass weight, muscle density, and reproductive seasonality (*PTK2B*, *FNIP1*, *EYA4*, *UBASH3B*, and *SPPL2B*);
- in Texel, genes associated with traits like bone density, meat yield, and hot carcass weight.

These differences have led to breed-specific genomic adaptations and economically important phenotypes.

## 6. Hybridization and Introgression: Genetic Exchange and Its Consequences

Hybridization, the reproduction of individuals from genetically distinguishable populations, occurs when wild animals and feral individuals from closely related domestic species share the same habitat [89]. While hybridization creates new genetic combinations, introgression is the process by which those new combinations, or specific beneficial traits, can become permanently established within a different population or species. Such events are well-documented, particularly on Mediterranean islands where mouflon and domestic or feral sheep populations coexist [12,48,90]. Genetic evidence, primarily derived from ovine medium-density SNP array genotypes, confirmed recent gene admixture between European mouflon and feral sheep [12,90]. Interestingly, extensive introgression has been detected also from wild sheep species (e.g., snow sheep, bighorn, thinhorn, and argali) into urial, Asiatic mouflon, and European mouflon [8]. Introgressed genes from snow sheep into Asiatic mouflon occurred before domestication (~13,924–11,580 years BP), while argali introgression coincided with domestication (~9972–10,180 years BP).

Genomic analyses have also revealed that introgression events from wild *Ovis* species have significantly contributed to the high degree of morphological differentiation observed among domestic sheep breeds, as well as to individual variation within breeds [91]. This demonstrates that introgression is a powerful evolutionary force. While it can threaten the genetic purity of endangered wild populations, it has historically been a significant driver of adaptive evolution and phenotypic diversification in domestic species. In domestic sheep, several introgressed genes (e.g., *PAPPA2*, *NR6A1*, *SH3GL3*, *RFX3*, *CAMK4*) were found to be associated with traits like wool, meat, milk production, immune response, and reproduction [8]. Domestic sheep genomes contain introgressed sequences from Iranian mouflon (6.8%), urial (1.0%), and argali (0.2%), with rare contributions from other wild species. The introgressed fragments are unevenly distributed among domestic sheep populations, showing geographic patterns [24]. In this study, the authors provided specific examples of introgressed genes and their associated traits, including:

- *RXFP2* haplotype, introgressed from Iranian mouflon. This region maps on chromosome 10 and it is associated with the spiral horn trait in domestic sheep. Local ancestry inference suggests that most haplotypes in polled breeds (e.g., Finnsheep, Gotland, Waggir, Afshari, East Friesian) are closely related to those of Iranian mouflon, indicating a possible origin of the polled phenotype from this wild species.
- *MSRB3* haplotype, introgressed from Argali. Located on chromosome 3, the MSRB3 gene encodes methionine sulfoxide reductase B3, which is strongly associated with ear morphology. Haplotype analysis revealed that a cluster closely related to argali haplotypes is prevalent in domestic sheep and linked to variations in ear size (e.g., small ears in Swiss White Alpine, Mossi, Diqing sheep; large/floppy ears in Waggir, Karakul, Duolang).
- *VPS13B* haplotype, introgressed from urial/mouflon. Strong introgression signals were detected in the *VPS13B* gene, with evidence of introgression from urial and a separate signal from mouflon. While its functional role in sheep is still being verified, observations in other species suggest *VPS13B* may influence facial shape, particularly nose morphology.

Table 2 reports the main introgressions events that have occurred from wild to domestic sheep breeds.

## 7. Implications for Conservation and Management

Hybridization is generally considered a threat to the genetic integrity and survival of established wild populations, primarily through mechanisms such as genetic swamping and outbreeding depression [91]. Hybrid individuals can survive and successfully reproduce, generating fertile offspring that perpetuate gene flow within the population [92]. Despite this, positive outcomes are also reported. Indeed, positive consequences such as adaptive introgression and hybrid speciation, although less common, can play a significant role in species adaptation and biodiversity [93]. Accordingly, hybridization should not be universally considered a threat to biodiversity. Its impacts should be assessed on a case-by-case basis, as it can, in some circumstances, serve as a conservation tool to increase genetic diversity and facilitate adaptation and may even be better for biodiversity than isolation [94,95].

From a phenotypic point of view, mouflon/sheep hybrids often exhibit intermediate characteristics; for example, differences observed in hybrids include basal horn circumference, horn ring consistency, tail length, and coat colors [7,96,97]. Constant monitoring of mouflon populations is recommended to maintain their genetic integrity and to avoid risks of genetic erosion through hybridization with domestic breeds and inbreeding depression [9,12,44,48]. Crucially, the removal of feral sheep from wild habitats and improved control of sheep raised in semi-wild conditions are essential conservation measures. Feral sheep pose the greatest threat to mouflon conservation by undermining genetic integrity, depleting resources, altering spatial behavior, and facilitating disease transmission [90]. Mediterranean islands, recognized for their rich biodiversity and as genetic reservoirs of unique genetic variants and species lineages, are particularly vulnerable and require targeted conservation efforts. The phenotypic changes in mouflon/sheep hybrids, such as horn shape, serve as visible markers of genetic exchange, alerting conservationists to the need for genetic monitoring and intervention [7,12,48,90]. However, phenotypic changes are not just outcomes of hybridization but can serve as crucial early warning signs for genetic erosion in wild populations, guiding targeted management actions. This underscores the importance of an integrated conservation approach, combining traditional ecological observation with advanced genomic techniques.

Phenotypic analysis and reporting of putative hybrids should then be followed by molecular investigations to confirm the hybridization status. In a study published in 2020, Somenzi et al. [97] identified and tested five AIM panels using supervised machine learning approaches to detect hybridization between Sardinian mouflon and domestic sheep. All AIM panels showed high accuracy in assessing ancestry proportions, with genome-wide panels performing better than chromosome-wide panels. They successfully discriminated pure mouflon, domestic sheep, and hybrids from mouflon and commercial sheep breeds such as Texel, Merino, and Lacaune in both simulated and real populations. In 2023, Šprem et al. [90] carried out a study on the European mouflon and feral sheep populations from the Adriatic island Dugi Otok, providing new guidelines to detect mouflon/sheep hybrids. These authors confirmed previous observations that hybrids exhibited intermediate phenotypic traits, such as greater horn circumference than mouflon but smaller than sheep, pronounced horn rings, shorter tails, and intermediate coat color. To confirm the diagnosis at the molecular level, they used ovine medium-density SNP array genotypes which allowed them to identify two hybrid individuals. Based on the ADMIXTURE v.1.3 analysis results, these hybrids had 82.2% and 94.1% of their genome attributable to feral sheep, with the remainder from mouflon ancestry. Using the NEWHYBRIDS software v.1.1, these two individuals were classified as backcross hybrids between F1 generation mouflon × feral sheep and pure feral sheep parents, with probabilities of 86.7% and 49.6%. Such an approach based on the use of molecular tools is critical for preserving the genetic integrity of mouflon and other endangered species.

## 8. Ongoing Debates and Future Research Directions

Despite significant advancements in understanding the phylogeny of mouflon and domestic sheep, several key unresolved questions persist, fueling ongoing scientific debates.

(a) True Wild Status of Mouflons, Systematic Classification, and Nomenclature

Although recent scientific evidence supports the idea of mouflon as true wild sheep, a fundamental debate continues over whether Mediterranean island mouflons are truly wild remnants of ancient populations or feral descendants of early domesticates. As a consequence, the correct classification of the Corsican and Sardinian mouflon, specifically whether they should be -designated *Ovis aries musimon* (a domestic sheep subspecies) or *Ovis gmelini musimon* (a wild subspecies), remains a critical unresolved question with direct implications for their conservation status and management strategies.

(b) Extent and Nature of Genetic Changes Attributable to Domestication

Questions persist about how much of the genetic makeup of current domestic sheep is directly due to human-mediated selection versus the retention of pre-existing genetic diversity from wild lineages.

(c) Discrepancies in Phylogenetic Reconstructions

Reconciling differences between phylogenies derived from mtDNA and nuDNA and fully understanding the implications of factors, such as incomplete lineage sorting and sex-biased dispersal, remain an active area of research.

(d) Specific Location, Timing, and Post-Domestication History

Many aspects of the exact location, timing, and subsequent history of sheep domestication and diffusion remain unresolved.

Addressing these complex questions necessitates continued and expanded research, focusing on several promising avenues, including:

- Expanded Genomic Sampling: WGS of a broader and more geographically representative range of wild mouflon populations, especially from isolated islands, coupled with ancient samples, would provide more comprehensive data to resolve phylogenetic ambiguities and delineate fine-scale population structure.
- Ancient DNA Studies: Further rigorous analysis of ancient DNA from Neolithic and Copper Age sheep can provide direct evidence of early haplogroup distribution and genetic diversity at the onset of domestication, offering a direct window into past genetic landscapes.
- Functional Genomics of Introgressed Regions: A deeper investigation into the functional roles of identified introgressed genes and their precise molecular mechanisms in shaping phenotypic traits would provide critical insights into the genetic architecture of domestication and adaptation.
- Longitudinal Studies of Hybrid Populations: Monitoring hybrid populations over multiple generations would allow for a comprehensive understanding of the long-term genetic and phenotypic consequences of introgression and backcrossing, informing more effective conservation strategies.
- Integrated Multi-Omics Approaches: Combining genomics with other “omics” technologies, such as transcriptomics, proteomics, and metabolomics, would enable a more holistic understanding of the physiological and developmental pathways affected by both domestication and introgression.
- Comparative Genomics across *Ovis* Species: Broader comparative studies across the entire *Ovis* genus are essential to clarify deeper evolutionary splits and resolve ancestral relationships, providing a more complete picture of sheep evolution.

## 9. Conclusions

The taxonomic debate over European mouflon, particularly its designation as a wild versus domestic subspecies, directly impacts conservation efforts. If classified as a domestic subspecies (*O. aries musimon*), conservation efforts might be deprioritized, as these populations could be perceived as less “wild” or genetically distinct. Conversely, reclassification as a wild subspecies (*O. gmelini musimon*) would elevate their conservation status, justifying interventions such as the removal of feral sheep and vigilant monitoring of genetic purity. This highlights that scientific classification is not merely descriptive but prescriptive, guiding policy and resource allocation for endangered or vulnerable populations. This underscores the critical link between fundamental research studies focused on phylogeny and taxonomy and applied conservation biology. Resolving these debates through robust genomic evidence is essential for effective management and protection of wild populations, particularly in vulnerable ecosystems such as Mediterranean islands. The trajectory of research suggests that future breakthroughs in understanding mouflon and sheep phylogeny will come from increasingly sophisticated genomic tools and analytical frameworks that can integrate diverse data types and resolve the subtle, complex genetic interactions that have shaped these species.

Based on the topics covered in this review, domestication was not a singular, isolated event but a continuous process involving complex genetic exchanges, including post-domestication introgression from various wild relatives [69]. It emphasizes the need for comprehensive genomic approaches to capture the full spectrum of genetic contributions. Future research, leveraging advanced genomic technologies and integrative approaches, promises to further unravel the complex evolutionary tapestry of *Ovis* species, providing deeper insights into their history and informing their ongoing conservation.

## Figures and Tables

**Table 1:** Overview of Key Mitochondrial DNA Haplogroups in *Ovis* Species.

HPG	Origin	Prevalence
A	Asian	Central Asian and some European domestic sheep.
B	European	Most domestic sheep and European mouflons.
C	Asia, Middle East, and Caucasus	Likely from Iranian Asiatic mouflon.
D	Caucasus and Turkey	Rarest, closest to *O. gmelini anatolica*.
E	Middle East and Caucasus	Key haplogroup of Iranian Asiatic mouflon.
X	Cyprus, Iran, and Turkey	Wild mouflon HPG, basal to domestic HPGs C and E.

**Table 2:** Genetic Introgression Events from Wild *Ovis* Species to Domestic Sheep.

Introgressed Region	Donor Species	Phenotypic Trait(s)	Specific Domestic Breeds	Significance
*RXFP2* (chr10)	Ir mouflon	Spiral horn trait/horn status (polled, sex-specific, horned)	Polled: Finnsheep, Gotland, Waggir, Afshari, East Friesian; Sex-specific: Chinese Merino, Ouessant, Barki; Horned: Oula, Prairie Tibetan, Valais Blacknose, Scottish Blackface.	Contributed to morphological differentiation, particularly horn development.
*MSRB3* (chr3)	Argali	Ear morphology (small, large/floppy)	Small ears: Swiss White Alpine, Mossi, Diqing; Large/floppy ears: Waggir, Karakul, Duolang.	Associated with variations in ear size and shape.
*VPS13B* (chr9)	Urial/mouflon	Facial traits (e.g., nose morphology)	Tibetan sheep (Oula, Prairie Tibetan, Valley Tibetan).	Potential influence on facial shape, contributing to breed-specific appearances.
(General)	*O. gmelini_2* (Kaboodan Island)	Body size, fat metabolism, local adaptation to hot and humid environments	Garut, Bangladeshi, Nellore, Sumatra sheep populations in South and Southeast Asia.	Contributed to adaptive traits for specific environments.

## Data Availability

No new data were created or analyzed in this study. Accordingly, data sharing is not applicable to this article.

## Abbreviations

The following abbreviations are used in this manuscript:

AIM	Ancestry Informative Marker
BCE	Before the Common Era
HPG	Haplogroup
NCC	Neural Crest Cell
SNP	Single-Nucleotide Polymorphism
TBXT	T-box Transcription Factor T Gene
WGS	Whole-Genome Sequencing
